# Decreased levels of serum zonulin and copeptin in chronic Hepatitis-B patients

**DOI:** 10.12669/pjms.35.3.144

**Published:** 2019

**Authors:** Mustafa Kerem Calgin, Yeliz Cetinkol

**Affiliations:** 1 *Dr. Mustafa Kerem Calgin, Medical Microbiology Department, Ordu University Faculty of Medicine, Ordu, Turkey*; 2 *Dr. Yeliz Cetinkol, Medical Microbiology Department, Ordu University Faculty of Medicine, Ordu, Turkey*

**Keywords:** Chronic Hepatitis B, Zonulin, Copeptin, Hepatitis Diagnosis

## Abstract

**Background & Objective:**

Liver and intestines are anatomically and physiologically linked. Zonulin is a protein modulating intercellular tight junctions and regulating intestinal permeability. Copeptin was studied as a marker of systemic circulation disorders in research about vasopressin and was associated with liver disease prognosis. Serum zonulin and copeptin levels were measured in patients with diagnosis of chronic hepatitis B (CHB) with the aim of easing antiviral treatment management in clinical applications and to investigate the association with normal population and viral load.

**Methods:**

Analysis included the serum of 30 CHB patients and 17 controls. HBV-DNA real-time PCR tests were completed. CHB patients were divided into three subgroups according to viral load in serum. Zonulin and copeptin levels were measured using ELISA kits.

**Results:**

Serum zonulin and copeptin levels were significantly low in CHB patients compared to controls (p<0.001). When CHB subgroups are investigated in terms of serum zonulin and copeptin levels, there was an inverse correlation observed with significant difference (p<0.01, p<0.05).

**Conclusion:**

The negative correlation between serum zonulin and copeptin with HBV-DNA load revealed in our study shows they may be used to monitor treatment. Zonulin and copeptin assays provide the possibility of developing new approaches to CHB diagnosis and monitoring.

## INTRODUCTION

Hepatitis B virus (HBV) infection is a significant global health problem causing chronic hepatitis, liver cirrhosis and hepatocellular carcinoma (HCC). The World Health Organization estimates that more than 350 million people are chronic HBV carriers.^1^ In people with this infection, monitoring the progression of the disease, treatment of chronic hepatitis and early diagnosis of linked diseases that may develop has a clinically important role. A variety of diagnostic tests have been developed to identify HBV infection. Serum viral load level is the most important marker used to begin antiviral treatment and assess treatment efficacy.^2^,^3^ Additionally, it is important to develop cheaper and more accessible methods for diagnosis and monitoring of chronic hepatitis B (CHB) infections.

The liver and intestine are anatomically and physiologically linked organs. The liver is linked to the small intestine through the bile duct and bile created in the liver is carried to the intestine in this way. Additionally, nearly all blood from the stomach and intestines passes through the liver. The human intestine hosts various species of bacterial assemblages called the intestine microbiota.^4^ Bacterial peptidoglycans, flagellins and other microbial metabolic by-products found in the intestines are known to worsen the clinical progression in patients with chronic liver diseases. Intestinal bacteria may penetrate the intestinal mucosa in cirrhosis patients by different mechanisms; however, the precondition for this is damage to the intestinal barrier causing increased mucosal permeability.^5^ The intestinal barrier is provided by the intercellular “tight junctions (TJs)” between intestinal mucosa cells. Faced with high resistance of enterocyte plasma membranes, dissolved material flow is via the paracellular route and TJs are the basic obstacle to this route. Previously TJs were considered to be static, but they are highly dynamic structures and there is evidence that they adapt to developmental, physiological and pathologic situations.^6^ In recent years, the physiologic modulators of TJs have been discovered. Zonulin is the only human protein regulating modulation of intercellular TJs and intestinal permeability known to date. Zonulin binds to receptors activating some intracellular pathways and as a result TJs are opened and permeability increases.^7^,^8^

To keep arterial blood pressure within normal limits for cirrhosis patients, clear activation forms in endogenous vasoconstrictor systems like the renin – angiotensin - aldosterone system, sympathetic nervous system and vasopressin (AVP).^9^ Copeptin, a 39 - amino acid glycopeptide, biochemically is released from the neurohypophysis within pioneer preprovasopressin together with AVP. Contrary to AVP, copeptin is a very stable and easy to measure, non-functional peptide. As a result, measurement of copeptin levels as a biomarker allowed the possibility of researching the role of AVP in clinical applications.^10^,^11^ Studies to date have shown that copeptin is not limited to its role in posterior hypophysis function, but simultaneously is a marker of systemic circulation disorder in liver diseases and is associated with prognosis.^12^,^13^

Zonulin and copeptin molecules have not been researched in HBV hepatitis to date. In this study, the serum zonulin and copeptin levels were measured in patients with diagnosis of CHB with the aim of easing antiviral treatment management in clinical applications and to investigate the association with viral load.

## METHODS

Serum from 30 CHB patients was included in the assessment with measurement of viral load in the molecular microbiology laboratory. Control serum was obtained from 17 clinic patients with no known history of chronic disease, no intestinal disease and negative for serum HBsAg, anti - HBs and anti - HCV titers. Serum was stored at - 40 °C until analysis. The study received permission from Ordu University clinical research ethics committee (2017 / 95).

### Real - Time PCRs

Quantitative HBV - DNA real - time PCR tests were completed using a COBAS AmpliPrep / COBAS Taqman 48 system (Roche, Branchburg, NJ, USA). Viral nucleic acids were extracted from 500 µl patient serum with a COBAS AmpliPrep automated extractor according to the manufacturer’s instructions. The measurement interval of the kit for HBV- DNA is 20 - 1.7 x 10^8^ IU/ml.

### ELISA

Serum copeptin measurements were completed with a Human Copeptin ELISA kit (Sunred Biological Technology®, Shanghai, People’s Republic of China) and serum zonulin measurements used a Human Zonulin ELISA kit (Sunred Biological Technology®, Shanghai, People’s Republic of China) according to the manufacturer’s instructions. Plates were read with a Synergy HT microplate reader (Biotek®, Winooski, VT, USA).

### Statistical Analysis

Normal distribution of data was checked with the Kolmogorov - Smirnov test, while homogeneity of group variance was checked with the Levene test. Variables abiding by assumptions were compared using the student T test for two groups and with the one - way ANOVA for more than two groups. Variables not abiding by assumptions were compared using the Mann - Whitney U test for two groups and the Kruskal - Wallis test for more than two groups. After the Kruskal - Wallis test, differing groups were determined with the Dunn test and results are represented by letters. Level of significance (α) for calculations and interpretation of results was taken as 5%. All statistical calculations were completed with the Statistical Package for the Social Sciences software 25.0 (IBM SPSS Statistics for Windows, Version 25.0. Chicago, IL: IBM Corp.).

## RESULTS

The study included a total of 47 serum samples (20 female, 27 male). Those with gastrointestinal disease and chronic disease other than hepatitis were excluded from the study. Serum was classified in two main groups; CHB (n = 30) and control group (n = 17). Serum from the CHB group was divided into three subgroups according to contained viral nucleic acid amounts (Table-I).

**Table-I:** Study groups.

	CHB (n=30) HBV-DNA levels (IU/ml)	Control (n=17)
Subgroup 1	0-1x10^2^ (n=10)	-
Subgroup 2	1x10^3^-1x10^5^(n=10)	-
Subgroup 3	1x10^6^-1x10^8^ (n=10)	-

CHB: Chronic Hepatitis B, IU/ml: International Units per millilitre, n: Number.

### Statistical results for CHB and control group

Student t test results for age found no statistically significant difference between the groups (p > 0.05). Mann - Whitney U test performed for zonulin and copeptin found the CHB group had statistically significantly lower values compared to the control group (p < 0.001) (Table-II).

**Table-II:** Characteristics of chronic hepatitis B patients and control group.

	CHB (n=30)	Control (n=17)	p value
Age (Mean±SD)	47.2±13.9	57.1±23.2	0.073^NS^
Zonulin (Median(IQR))	3.8(4.5)	12.7(2.6)	0.000^***^
Copeptin (Median(IQR))	1.08(1.04)	3.7(0.8)	0.000^***^

CHB: Chronic Hepatitis B, SD: Standard deviation, IQR: Interquartile Range, n: Number

NS: Statistically non-significant (p>0.05)

*** : Statistically significant (p<0.001).

### Statistical Results for CHB subgroups

Variance analysis for the age variable found the differences between the subgroups were not statistically significant (p > 0.05). Kruskal - Wallis test results for zonulin found the difference between subgroups was statistically significant (p < 0.05). The Dunn multiple comparison test performed with the aim of determining the differing groups found a statistically significant difference between subgroup one and subgroup three (p < 0.05). Subgroup two was not found to be statistically different from both subgroup one and subgroup three (p > 0.05). Kruskal - Wallis test results for copeptin found no statistically significant difference between the groups (p > 0.05) (Table-III).

**Table-III:** Characteristics of chronic hepatitis B subgroups.

	Subgroup1 n=10	Subgroup2 n=10	Subgroup3 n=10	p value
Age (Mean±SD)	51.2±13.7	40.3±13.6	50.2±12.7	0.159^NS^
Zonulin (Median(IQR))	5.3(5.2)^A^	4.7(4.4)^AB^	1.6(3.1)^B^	0.042*
Copeptin(Median(IQR))	1.4(1.4)	1.1(0.9)	0.6(0.9)	0.170^NS^

SD: Standard deviation, IQR: Interquartile Range, n: Number NS: Statistically non-significant (p>0.05)

* Statistically significant (p<0.05)AB: The differences between groups without a common letter were statistically significant (p<0.05).

The Spearman coefficients calculated with the aim of determining the correlation between the DNA amounts and serum zonulin and copeptin amounts in the subgroups are given in Table-IV. The correlation coefficient between subgroup DNA amounts and serum zonulin levels was calculated as -0.510 and was statistically significant (p < 0.01). As serum DNA amounts increased, there was a reduction in zonulin amounts. The correlation coefficient for subgroup DNA amounts with serum copeptin levels was calculated as -0.415 and was found to be statistically significant (p < 0.05). As the serum DNA amount increased, there was a reduction in copeptin amounts.

**Table-IV:** Correlation coefficients between subgroups, zonulin and copeptin.

		Zonulin	Copeptin
Subgroup	r	-0.510	-0.415
	p-value	0.004*	0.023**

* : Statistically significant (p<0.01)

** : Statistically significant (p<0.05).

## DISCUSSION

In recent years, zonulin, a peripheral marker of TJ and intestinal permeability, has begun to be researched in many areas of medicine including sepsis, autoimmune diseases, malignancy and central nervous system disorders.^6^ Zonulin levels have been identified to be high in the patient group compared to controls in some studies, while low levels have been identified for some diseases.^14^-^16^ Copeptin the stable product of AVP, reflecting the degree of hemodynamic disorder in liver cirrhosis, is a molecule with potential as a prognostic marker. New studies have shown it is a new and reliable biological marker for disease progression, clinical decompensation and prognosis in cirrhosis patients, reflecting the circulation function disorder occurring in advanced cirrhosis patients. Studies comparing decompensated cirrhosis patients with compensated cirrhosis patients have observed significantly high levels of plasma copeptin.^9^ This study is the first research to investigate serum zonulin and copeptin levels in CHB patients and to compare with viral load.

The intestinal microbiota forms a complicated ecologic system playing a significant role in human immunology, nutrition and pathologic processes.^17^,^18^ Much research has proposed an important role for intestinal microbiota changes in the initiation and progression of liver diseases. When the balance of the gastrointestinal ecosystem is disrupted, bacteria may change the intestinal permeability.^19^During some pathogenic bacterial infections, increases in zonulin secretion have been observed in the small intestine.^20^ Chou et al. showed that balanced intestinal microbiota plays an important role in the regulation of host immunity required for clearance of HBV from the body.^21^ Additionally, variations in intestinal flora of CHB patients have been shown in a variety of studies and the specific species of intestinal commensal bacteria may play a protective or pathogenic role in development of diseases associated with HBV.^4^ In our study, it is considered that the intestinal permeability of CHB patients may be reduced compared to the normal population due to flora changes. Additionally, identification of high intestinal permeability in CHB patients with low viral load compared to patients with high viral load leads to the consideration that the regulation functions of intestinal microbiota may be disrupted in these patients. Our study has some limitations such as not analyzing intestinal microflora or performing intestinal permeability tests.

Zonulin secretion reduces the function of the intestinal barrier.^20^ Examined from another aspect, the aim of the stronger intestinal barrier functions in CHB patients compared to the control group and reduction in mucosal permeability developing may be to prevent the transition of toxic bacterial products into the bloodstream. Attempts to reduce the transition of toxic products into blood circulation may cause the identification of low levels of copeptin, a marker of systemic circulation disorder observed to increase especially in cirrhosis, in patients. Additionally, many non-infective HbsAg particles form in CHB patients. It is considered that the increase observed in barrier functions compared to the control group may be due to the interaction of these particles with intestinal epithelium. This situation shows that intestinal epithelial cells may enter interactions with viral particles.

Viral load, used with the aim of assessing antiviral treatment, shows active viral replication. Disease develops as a result of replication of viral hepatitis and as a result it is known that histologic damage may develop.^22^ Additionally, there are studies showing that HBV - DNA levels in CHB infection do not indicate the severity of liver inflammation or fibrosis.^23^ Though a threshold serum HBV - DNA level associated with progression of liver disease has not been found, high viral load (> 1 x 10^5^ copies/mL) increases the risk of mortality and HCC associated with the liver.^24^ Some studies have shown that low viremia (serum HBV - DNA ≤1 x10^5^ copies/mL) may cause liver cirrhosis and carries increased risk of HCC development.^22^ Though serum HBV - DNA concentration is not a definite marker for CHB, it may be used to predict the development of liver disease. In our study, patients with low serum HBV - DNA load (subgroup 1) had high zonulin amounts, showing intestinal permeability increased in patients with low viral loads. The elevation in copeptin amounts identified in the same patients shows simultaneous disruption of systemic circulation. Our findings support the opinion that risk of cirrhosis and HCC development may increase for CHB patients with low viremia.

## CONCLUSION

The negative correlation of serum zonulin and copeptin molecules with HBV - DNA load revealed in our study shows that the biomolecules of zonulin and copeptin may be used for treatment monitoring in these patients. These results may allow the development of new approaches to diagnosis and monitoring of HBV infections. Additionally, the relationships between zonulin and copeptin metabolism and chronic viral hepatitis infections is very complicated. To support these results, there is a need for more comprehensive studies with the aim of assessing long term effects on patients, and understanding the relationships between viral load with intestinal permeability and systemic circulation disorders in chronic viral hepatitis patients.

### Authors’ Contribution

**MKC** conceived, designed and did statistical analysis & editing of manuscript.

**MKC and YC** did data collection and manuscript writing.

**YC** did review and final approval of manuscript.
